# Image Quality and Diagnostic Performance of Accelerated 2D Hip MRI with Deep Learning Reconstruction Based on a Deep Iterative Hierarchical Network

**DOI:** 10.3390/diagnostics13203241

**Published:** 2023-10-18

**Authors:** Judith Herrmann, Saif Afat, Sebastian Gassenmaier, Gregor Koerzdoerfer, Andreas Lingg, Haidara Almansour, Dominik Nickel, Sebastian Werner

**Affiliations:** 1 Department of Diagnostic and Interventional Radiology, Eberhard Karls University Tuebingen, Hoppe-Seyler-Strasse 3, 72076 Tuebingen, Germany; 2 MR Applications Predevelopment, Siemens Healthcare GmbH, Allee am Roethelheimpark 2, 91052 Erlangen, Germany

**Keywords:** magnetic resonance imaging, deep learning reconstruction, image processing, diagnostic imaging, hip

## Abstract

Objectives: Hip MRI using standard multiplanar sequences requires long scan times. Accelerating MRI is accompanied by reduced image quality. This study aimed to compare standard two-dimensional (2D) turbo spin echo (TSE) sequences with accelerated 2D TSE sequences with deep learning (DL) reconstruction (TSE_DL_) for routine clinical hip MRI at 1.5 and 3 T in terms of feasibility, image quality, and diagnostic performance. Material and Methods: In this prospective, monocentric study, TSE_DL_ was implemented clinically and evaluated in 14 prospectively enrolled patients undergoing a clinically indicated hip MRI at 1.5 and 3T between October 2020 and May 2021. Each patient underwent two examinations: For the first exam, we used standard sequences with generalized autocalibrating partial parallel acquisition reconstruction (TSE_S_). For the second exam, we implemented prospectively undersampled TSE sequences with DL reconstruction (TSE_DL_). Two radiologists assessed the TSE_DL_ and TSE_S_ regarding image quality, artifacts, noise, edge sharpness, diagnostic confidence, and delineation of anatomical structures using an ordinal five-point Likert scale (1 = non-diagnostic; 2 = poor; 3 = moderate; 4 = good; 5 = excellent). Both sequences were compared regarding the detection of common pathologies of the hip. Comparative analyses were conducted to assess the differences between TSE_DL_ and TSE_S_. Results: Compared with TSE_S_, TSE_DL_ was rated to be significantly superior in terms of image quality (*p* ≤ 0.020) with significantly reduced noise (*p* ≤ 0.001) and significantly improved edge sharpness (*p* = 0.003). No difference was found between TSE_S_ and TSE_DL_ concerning the extent of artifacts, diagnostic confidence, or the delineation of anatomical structures (*p* > 0.05). Example acquisition time reductions for the TSE sequences of 52% at 3 Tesla and 70% at 1.5 Tesla were achieved. Conclusion: TSE_DL_ of the hip is clinically feasible, showing excellent image quality and equivalent diagnostic performance compared with TSE_S_, reducing the acquisition time significantly.

## 1. Introduction

Hip pain is common in adults of all ages and activity levels [1]. A standing radiograph is the recommended initial imaging test. Still, magnetic resonance imaging (MRI) has evolved into an essential tool in modern musculoskeletal imaging, particularly valued for its superior soft tissue contrast and lack of ionizing radiation. In hip joint imaging, MRI is useful for identifying a range of pathologies, e.g., labral tears, cartilage lesions, and tendinopathies.

For a standard hip MRI, the German Radiological Society recommends the following sequences: coronal or axial fat-suppressed T2- or PD-weighted TSE of both hips for an initial overview; coronal T1-weighted and fat-suppressed PD-weighted, axial oblique PD-weighted with or without fat-suppression, sagittal PD-weighted, and optional sagittal True-FISP Water Excitation for cartilage imaging [2]. The standard protocol at our institution has an acquisition time of approximately 23 min and includes a coronal turbo inversion recovery magnitude (TIRM), a coronal T1-weighted TSE, and an axial T2-weighted TSE of the pelvis. The respective hip joint is examined using coronal and axial oblique fat-suppressed PD-weighted TSE and a 3D Double Echo Steady State (DESS). Turbo spin echo (TSE) sequences have become fundamental in routine musculoskeletal MRI examinations given their excellent trade-off between image quality and acquisition time [3]. However, there remains room for improvement. Numerous advanced acceleration methods exist, such as three-dimensional isotropic imaging, parallel imaging (PI), simultaneous multi-slice acquisition (SMS), compressed sensing (CS)-based sampling, and synthetic MRI. These techniques can be combined and potentially accelerate joint MRI acquisition times by up to eightfold [4,5,6,7,8,9,10,11,12,13,14,15,16,17].

On the other hand, in recent years, deep learning (DL)-based reconstruction techniques have rapidly gained popularity, harnessing the power of artificial intelligence and showing promise in further reducing scan times and possibly enhancing image quality [18,19,20,21,22,23,24,25,26,27]. DL techniques leverage artificial intelligence algorithms to reconstruct images from undersampled data, making them an exciting area of exploration.

To the best of our knowledge, there is a lack of studies comparing conventional and DL-reconstructed 2D TSE sequences of the hip joint considering factors such as acquisition time, image quality, visualization of anatomy, and diagnostic performance. This study aims to fill this gap, potentially providing valuable insights for optimizing MRI protocols for hip joint imaging.

The objective of our study is to evaluate the efficacy of using a deep learning (DL) reconstruction for turbo spin echo (TSE) sequences in hip MRI in comparison with conventional TSE sequences. The underlying hypothesis is that the use of DL for image reconstructions of accelerated hip MRI can substantially decrease examination times while maintaining both image quality and diagnostic confidence related to the specific anatomy of the hip.

## 2. Material and Methods

### 2.1. Study Design

For this prospective single-center study, institutional review board approval and written informed consent were obtained from all participants. Participation in the study was voluntary. All study procedures were conducted per the ethical standards laid down in the 1964 Declaration of Helsinki and its later amendments.

Adult patients who underwent clinically indicated hip MRI between October 2020 and May 2021 were prospectively included in this study. Inclusion criteria were informed consent to participate in the study and a complete imaging protocol, including standard TSE sequences (TSE_S_) and DL-accelerated TSE sequences (TSE_DL_). Exclusion criteria were insufficient image quality pertaining to factors not inherent to the MRI sequence or reconstruction technique (for example, movement artifacts) and orthopedic hardware in the pelvis and hip region, as well as lack of consent for study participation. A final sample of 14 consecutive patients (mean age, 46 ± 13 years; range, 23–66 years; 7 males, 7 females) was included in this study (Table 1).

All included patients were examined with clinically used 1.5 T and 3 T scanners (MAGNETOM Aera, MAGNETOM Avanto^fit^, MAGNETOM Skyra, MAGNETOM Prisma^fit^, and MAGNETOM Vida; all from Siemens Healthcare, Erlangen; Germany). The institution’s standard protocol for hip imaging consists of a T1-weighted TSE in the coronal plane, a T2-weighted TSE in the axial plane of the pelvis, and a proton density (PD)-weighted TSE in the axial oblique and coronal plane with fat saturation, followed by a coronal T2 TIRM of the pelvis and a sagittal 3D DESS of the hip. In each patient, first, the institution’s standard protocol using generalized autocalibrating partial parallel acquisition reconstruction (GRAPPA) was employed (TSE_S_). Second, each TSE sequence was again acquired undersampled and using a DL-based reconstruction (TSE_DL_). Example acquisition parameters are given in Table 2.

For the TSE_DL_, the undersampling method and the research DL reconstruction technique used in this study were already described in detail in prior studies [28,29]. To accelerate the data acquisition, a conventional undersampling pattern known as PI was used [28,30]. Depending on the sequence, parallel imaging acceleration factors between 2 and 4 were used. K-space data, precomputed coil sensitivity maps, and a bias field for image homogenization were inserted into the variational network, consisting of multiple cascades, each made up from data consistency using a trainable Nesterov Momentum followed by convolutional neural network (CNN)-based regularization [22].

The reconstruction was trained on volunteer acquisitions using conventional TSE protocols independently of the data acquired in this study. About 10,000 slices were acquired from volunteers using clinical 1.5 T and 3 T scanners (MAGNETOM scanners, Siemens Healthcare, Erlangen, Germany). Using representative protocols for the respective body regions, fully sampled high-resolution acquisitions were performed in various anatomies, such as the head, pelvis, and knee. The training data included different image contrasts, orientations, body regions, and resolutions. The training was implemented in PyTorch and performed on a GPU cluster NVIDIA Tesla V100 (32 GB of memory) GPU. For deployment in the clinical setting, the trained network was converted for prospective use in a proprietary C++ inference framework and integrated into the scanners’ reconstruction pipeline.

Siemens Healthcare, Erlangen, Germany, provided the research DL reconstruction application, but complete control of patient data was with the authors.

### 2.2. Image Evaluation

Two radiologists independently assessed the images of the TSE datasets, and both readers were blinded toward reconstruction type, patient data, clinical and radiological reports, and each other’s assessments. TSE_DL_ and TSE_S_ sequences were separated, resulting in 28 datasets. Reading sessions were randomly conducted at a dedicated workstation (GE Healthcare Centricity™ PACS RA1000, Milwaukee, WI, USA). Images were evaluated regarding image quality, artifacts, noise, edge sharpness, and diagnostic confidence using a 5-point Likert scale (5 = best). Reading scores were considered sufficient when reaching ≥ 3. In addition, TSE_S_ and TSE_DL_ were evaluated for the quality of the delineation of the following anatomical structures using a 5-point Likert scale (5 = best): labrum, iliofemoral ligament, ligamentum teres, psoas tendon, abductor tendons, and cartilage. The presence of lesions of these same anatomical structures (labrum, iliofemoral ligament, ligamentum teres, psoas tendon, abductor tendons, and cartilage), as well as the presence of degeneration, bursitis, joint effusion, or fractures, was classified by both readers in a binary manner (present/absent).

If there were discrepancies between the readers, a consensus reading was enclosed to define false positive and false negative findings.

### 2.3. Quantitative Analysis

Quantitative analysis of the signal-to-noise ratio (SNR) was performed by measuring the signal intensities (SIs) and standard deviation of the mean as an indicator of noise using the following formula:

$$SNR=\frac{SI}{standard\;deviation}$$

On the coronal slices of the PD-weighted TSE sequences, a region of interest of 1 cm^2^ was manually drawn in the adductor muscle and the femoral neck at the exact same location upon TSE_S_ and TSE_DL_. Large vessels and focal lesions were avoided in the measurements.

### 2.4. Statistical Analysis

Statistical analyses were performed using SPSS version 28 (IBM Corp, Armonk, NY, USA). Descriptive statistics were used to summarize participants’ demographics and clinical characteristics. The mean, median, and interquartile range are reported for ordered categorical variables, and the mean and standard deviation are reported for continuous variables. A paired-sample Wilcoxon signed-rank test was used to compare the sequences in terms of the image quality scores from each reader. Inter- and intra-reader agreement was used to assess weighted Cohen’s κ, both with 95% confidence intervals, and interpreted as follows: 0.20 or less, poor agreement; 0.21–0.40, fair agreement; 0.41–0.60, moderate agreement; 0.61–0.80, substantial agreement; and greater than 0.80, almost perfect agreement.

## 3. Results

Among 46 eligible participants, a final sample of 14 participants (mean age 46 ± 13; range 23–66 (years); 7 males, 7 females) were prospectively included in this study. Eight examinations were performed on 1.5 T and six on 3 T regardless of diagnosis, current treatment, first examination, or follow-up (Table 1).

Using the data from the example hip MRI protocol used at our institution (Table 2), we can see that a typical standard 3 Tesla hip examination without contrast administration and including the standard TSE sequences, as well as the coronal T2 TIRM of the pelvis and the additional sagittal 3D DESS, takes 23:10 min. The analogous protocol using the DL-reconstructed TSE sequences takes only 16:00 min, signifying a scan time reduction of approximately 31%. Taking only the TSE sequences into account, the acquisition time is reduced by 53% from 13:32 min to 06:22 min. At 1.5 Tesla, the respective scan time reductions are 39% (23:16 min vs. 14:05 min) for the entire protocol and 70% (13:08 min vs. 3:57 min) for the TSE sequences.

### 3.1. Image Quality

The inter-reader agreement was substantial to almost perfect, with values between 0.609 and 1.000. Because of the good inter-reader reliability, only the results of reader 1 are provided in the following paragraph.

The image quality of TSE_DL_ was rated superior (median 4, IQR 4-4) compared with TSE_S_ (median 4, IQR 3-4, *p* = 0.008). The extent of artifacts was rated similarly between TSE_DL_ and TSE_S_ (median 4, IQR 3-4, *p* > 0.05). The extent of noise was rated significantly lower in TSE_DL_ (median 4, IQR 4-4) compared with TSE_S_ (median 3, IQR 3-3, *p* < 0.001). Edge sharpness was rated significantly superior in TSE_DL_ (median 45, IQR 4-4) compared with TSE_S_ (median 3, IQR 3-4, *p* = 0.003). No difference was found regarding the diagnostic confidence of either reconstruction method (median 4, IQR 4-, *p* > 0.05).

A summary of the qualitative image analysis, including the results by reader 2 and the inter-reader agreement (Cohen’s κ), is provided in Table 3.

**Figure 1:**
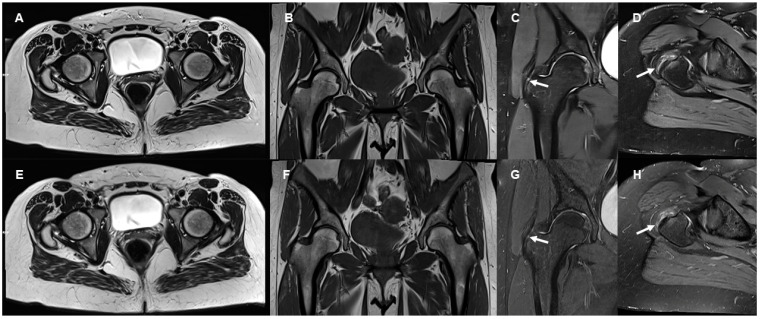
Examples of images comparing TSE_DL_ (**A**–**D**) and TSE_S_ (**E**–**H**) at 3 Tesla are provided. A 45-year-old female patient with progressive hip pain for 8–12 weeks in the trochanteric region; the MR images provided the diagnosis of abductor tendinopathy. The axial DL-reconstructed T2-weighted TSE image of the pelvis (**A**) exhibits enhanced sharpness while maintaining a similar overall appearance to the standard reconstruction (**E**). Similarly, the DL-reconstructed coronal T1-weighted TSE image of the pelvis (**B**) demonstrates a slight increase in sharpness and reduced artifacts compared with the standard TSE (**F**). In the DL-reconstructed coronal PD-weighted fat-suppressed TSE image of the right hip, the DL reconstruction exhibits noticeably reduced noise in comparison with the standard reconstructions (**G**); the small abductor tendon lesion (arrows) is slightly more visible in the DL reconstruction. The abductor tendinopathy with surrounding edema is well demonstrated in both the DL-reconstructed axial oblique PD-weighted image (**D**) and the corresponding standard reconstruction (**H**) even though there are some visible banding artifacts in the TSE_DL_ image.

**Figure 2:**
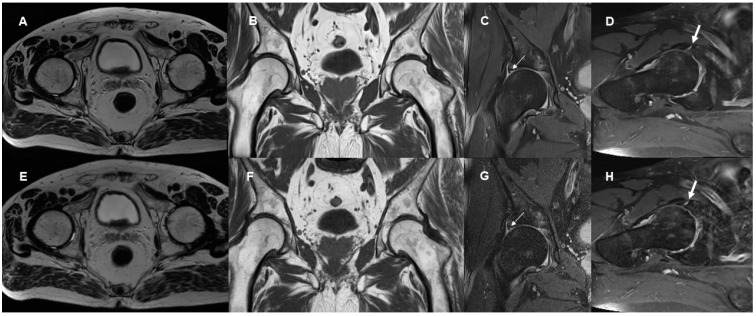
Image examples contrasting TSE_DL_ (**A**–**D**) and TSE_S_ (**E**–**H**) at 1.5 Tesla. A 67-year-old male patient with right-sided hip pain and suspected labral lesion. The axial DL-reconstructed T2-weighted TSE image of the pelvis (**A**) demonstrates increased sharpness compared with the standard reconstruction (**E**) while maintaining a similar image impression. A comparable increase in sharpness can be seen in the DL-reconstructed coronal T1-weighted TSE of the pelvis (**B**) compared with the standard TSE (**F**). In the coronal PD-weighted fat-suppressed TSE images of the right hip, the DL reconstruction (**C**) shows visibly less noise than the standard reconstruction (**G**); the lesion at the base of the superior labrum (arrows) appears slightly sharper in the DL-reconstructed image. In the sagittal PD-weighted fat-suppressed TSE images, noise and artifacts are reduced in the DL-reconstructed image (**D**) compared with the standard TSE (**H**); the labral lesion (arrows) is depicted well in both images.

**Figure 3:**
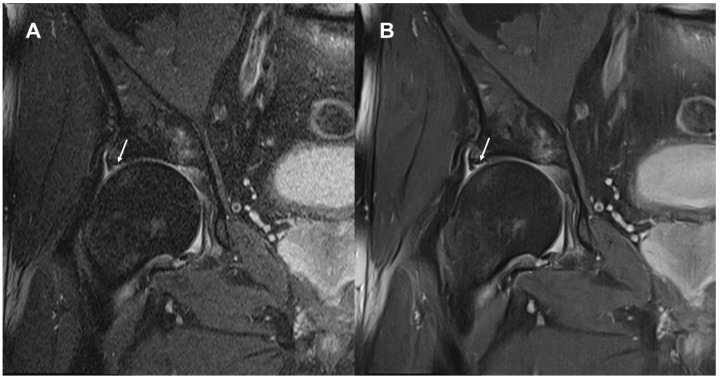
Image examples of the right hip at 1.5 Tesla. A 65-year-old male patient with hip pain and suspected labral lesion. Image (**A**) shows the standard coronal fat-suppressed PD-weighted TSE (TSE_S_). Image (**B**) depicts the DL-reconstructed coronal fat-suppressed PD-weighted TSE (TSE_DL_). Noise is visibly reduced in the TSE_DL_ image, although it shows the characteristic banding artifacts manifesting as thin vertical lines, more visible at the femoral head. Edge sharpness is also increased in the DL-reconstructed image. The small lesion at the base of the labrum and the adjacent small cartilage lesion (arrows) are depicted slightly sharper in the TSE_DL_ image.

**Figure 4:**
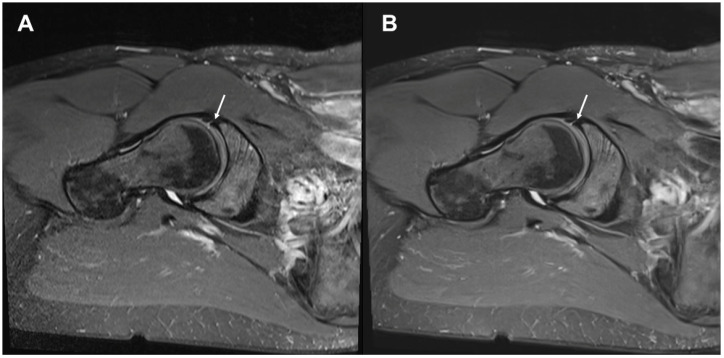
Image examples of the right hip at 1.5 Tesla. A 44-year-old female patient with impingement syndrome. Image (**A**) shows the standard axial oblique fat-suppressed PD-weighted TSE (TSE_S_). Image (**B**) depicts the DL-reconstructed axial oblique fat-suppressed PD-weighted TSE (TSE_DL_). Noise is visibly reduced in the TSE_DL_ image. There are only very slight banding artifacts in the partially depicted pelvic cavity. A thin hyperintense line at the base of the anterior labrum (arrows) is consistent with a tear, which is equally discernable in both images.

Image examples contrasting TSE_S_ and TSE_DL_ are provided in Figure 1, Figure 2, Figure 3 and Figure 4.

### 3.2. Delineation of Anatomic Structures and Internal Derangement

No statistically significant difference was found concerning the delineation of anatomical structures (labrum, iliofemoral ligament, ligamentum teres, psoas tendon, abductor tendons, and cartilage) between TSE_S_ and TSE_DL_. There was no clinically relevant difference regarding the detection of pathologic lesions between TSE_S_ and TSE_DL,_ and no difference was found in the detection of lesions of the labrum, the iliofemoral ligament, the ligamentum teres, the psoas tendon, the abductor tendons, or the cartilage (Table 4). No difference was found between the readers and the two reconstruction types concerning the detection of degeneration, bursitis, or fractures. In one patient, the presence of joint effusion was rated differently by reader 1 (TSE_S_: absent and TSE_DL_: present; see Figure 3). A consensus reading of this dataset was enclosed. Both readers confirmed that there was no relevant joint effusion. The different evaluation was due to the subjective assessment since there is no quantitative threshold between physiological fluid and joint effusion.

### 3.3. Quantitative Image Analysis

The quantitative measurements of noise were significantly lower in TSE_DL_ compared with TSE_S_ both in muscle (TSE_DL_: 9.94 ± 4.84, TSE_S_: 16.98 ± 5.03, *p* < 0.001) and bone (TSE_DL_: 18.36 ± 11.67, TSE_S_: 22.04 ± 9.40, *p* = 0.008). The quantitative measurements of SNR were significantly higher in TSE_DL_ compared with TSE_S_ both in muscle (TSE_DL_: 16.41 ± 8.07, TSE_S_: 8.39 ± 2.11, *p* < 0.001) and bone (TSE_DL_: 6.81 ± 5.66, TSE_S_: 4.63 ± 2.51, *p* = 0.008).

## 4. Discussion

This study aimed to evaluate the clinical applicability of a DL reconstruction algorithm for the prospectively undersampled TSE imaging of the hip. Our data reveal that TSE_DL_ shows superior image quality compared with TSE_S_ and is on par in various parameters, such as overall artifacts, the delineation of anatomical structures, pathology detection, and diagnostic confidence. Furthermore, TSE_DL_ offers less noise and enhanced edge sharpness alongside an impressive reduction in examination time of 63%.

To the best of our knowledge, the only study similar to ours was conducted by Koch et al. [21]. Their investigation regarded hip and shoulder MRIs of 54 patients and included 22 hip MRIs. They contrasted a DL reconstruction method equipped with two settings for noise reduction (reduction of 50% or 75%) with conventional reconstructions. They subjectively rated anatomic and pathologic conspicuity and overall image quality on a three-point scale. Their findings revealed superior performance by both DL settings over the conventional reconstruction, with the 75% noise reduction setting outdoing its 50% counterpart. Interestingly, although the cumulative performance was best at the 75% setting, the consensus among raters regarding the quality of the two DL settings varied. Quantitative evaluations confirmed that both denoising settings of the DL reconstruction improved relative edge sharpness, signal-to-noise ratio (SNR), and contrast-to-noise ratio (CNR), again with the 75% setting surpassing the 50% setting. There are, however, some distinct differences in their methodology. For instance, the authors relied solely on 3 T scanners and excluded 1.5 T ones. Furthermore, their reconstructions were based on fully sampled k-space data. In contrast, our DL algorithm incorporated prospective undersampling patterns known as parallel imaging. For our conventional images, we used GRAPPA reconstruction. Lastly, they restricted their image analysis to fat-suppressed intermediate-weighted sequences.

We observed no significant discrepancy in the quality of anatomical structure delineation between TSE_S_ and TSE_DL_. Comparable results were reported in a study by Kim et al. [18], which assessed knee MRIs for various conditions, including medial and lateral meniscus tears, anterior cruciate ligament tears, bone marrow edema, and high-grade cartilage defects. Meanwhile, another study by Foreman et al. evaluated ankle MRIs with DL-accelerated compressed sensing for abnormalities in several anatomical structures. They achieved an increased SNR with a 63% reduction in acquisition time, yet the depiction of structures was significantly lower, barring the tibionavicular ligament [19].

Other advanced acceleration techniques, such as parallel imaging (PI) and simultaneous multi-slice acquisition (SMS), have already been established. Generally, PI decreases acquisition time by sampling a limited number of phase-encoding steps, for example, every second or third step. However, this could result in a loss in SNR, which inversely corresponds to the square root of the acceleration factor. Conversely, SMS can increase specific absorption rate values because of simultaneous radiofrequency pulses [31]. PI and SMS can also suffer from aliasing and reconstruction artifacts with higher acceleration factors [16,32]. Compressed sensing (CS) involves nonlinearly undersampling phase-encoding information and recovering missing data during image reconstruction, which can be more time-consuming than PI-based reconstructions. However, recent software and hardware performance advances have mitigated this issue [31].

It is essential to consider the limitations of our study when interpreting the results: The restricted sample size and the single-center study design may potentially impact the broader applicability of our findings. Furthermore, there were slight differences in the acquisition parameters for TSE_S_ and TSE_DL_ between the 1.5 T and 3 T scanners. While the readers were blinded toward the imaging sequences, distinct visual characteristics may have allowed them to discern the reconstruction method used. Consequently, we cannot completely rule out the influence of individual bias on the results. The study utilized MRI scanners from only one manufacturer, which is another limiting factor.

Looking ahead, it would be beneficial to employ the DL-reconstruction method in a more extensive patient group, harmonize protocols for comparison, and analyze diagnostic performance in specific pathologies.

To conclude, the results of our study comparing TSE_S_ and TSE_DL_ suggest equal delineation of anatomical structures, pathology detection, and diagnostic confidence. Interestingly, TSE_DL_ seems to have the potential for reduced image noise and improved edge sharpness. Given that TSE_DL_ can decrease the acquisition time of the TSE sequences used in our standard protocol by up to 53% to 70%, depending on the magnetic field strength, TSE_DL_ holds significant clinical promise for hip imaging to enhance patient comfort and increase patient throughput.

## Figures and Tables

**Table 1:** Participants’ characteristics.

Characteristics	Patients
Total (male/female), *n*	14 (7/7)
Age, mean ± SD (range), y	total: 46 ± 13 (23–66)
Tesla, *n*	3 Tesla: 6 1.5 Tesla: 8

SD indicates standard deviation; y, years; *n*, number.

**Table 2:** Example MRI acquisition parameters.

	Sequence	FS	Area	Orientation	Contrast Agent	TA	Slices	Slice Thickness	FOV	TE	TR	FA	AV
3 T	T1 TSE_S_		pelvis	coronal	native	3:57	30	3	247 × 360	21	522	140	2
	T1 TSE_DL_		pelvis	coronal	native	1:48	30	3	247 × 360	25	480	160	1
	T2 TSE_S_		pelvis	axial	native	4:52	30	3	360 × 360	124	6230	140	3
	T2 TSE_DL_		pelvis	axial	native	2:38	30	3	360 × 360	124	3800	140	2
	PD TSE_S_	FS	hip	coronal	native	1:52	28	3	200 × 200	42	3410	150	1
	PD TSE_DL_	FS	hip	coronal	native	0:56	28	3	200 × 200	41	3300	160	1
	PD TSE_S_	FS	hip	axial oblique	native	2:51	30	3	200 × 200	42	3650	150	1
	PD TSE_DL_	FS	hip	axial oblique	native	1:00	30	3	200 × 200	41	3540	160	1
	T2 TIRM	FS	pelvis	coronal	native	3:22	30	3	247 × 360	74	5650	150	2
	3D T2 DESS	FS	hip	sagittal	native	6:16	80	1	170 × 170	9	25	25	2
1.5 T	T1 TSE_S_	FS	pelvis	coronal	native	3:48	28	3	226 × 329	21	492	150	2
	T1 TSE_DL_	FS	pelvis	coronal	native	0:52	28	3	226 × 329	18	619	150	1
	T2 TSE_S_		pelvis	axial	native	4:39	30	3	330 × 330	119	5940	140	3
	T2 TSE_DL_		pelvis	axial	native	0:49	30	3	330 × 330	119	4870	140	1
	PD TSE_S_	FS	hip	coronal	native	1:51	28	3	200 × 200	40	3380	150	1
	PD TSE_DL_	FS	hip	coronal	native	0:56	28	3	200 × 200	38	3260	150	1
	PD TSE_S_	FS	hip	axial oblique	native	2:50	30	3	200 × 200	40	3620	150	1
	PD TSE_DL_	FS	hip	axial oblique	native	1:20	30	3	200 × 200	38	3490	150	1
	T2 TIRM	FS	pelvis	coronal	native	3:52	30	3	240 × 350	70	5080	150	2
	3D T2 DESS	FS	hip	sagittal	native	6:16	80	1	170 × 170	9	25	25	2

FS indicates fat saturation; TA, time of acquisition; FOV, field of view; TE/TR, echo time/repetition time (ms); FA, flip angle (degree); AV, averages; AF, acceleration factor; T, Tesla; T1, T1-weighted; T2, T2-weighted; TSE, turbo spin echo; S, standard; DL, deep learning; PD, proton density.

**Table 3:** Results of Likert scale ratings for general image quality items and delineation of anatomical structures (presented as median with interquartile range in parentheses) and inter-reader agreement of standard TSE (TSE_S_) and deep learning-reconstructed TSE (TSE_DL_).

	Reader 1			Reader 2			Cohen‘s κ	
Item	TSE_S_	TSE_DL_	*p*-Value	TSE_S_	TSE_DL_	*p*-Value	TSE_S_	TSE_DL_
Image Quality	3 (3-4)	4 (4-4)	0.008	4 (3-4)	4 (4-4)	0.020	0.857	0.650
Artifacts	4 (3-4)	4 (3-4)	0.564	4 (3-4)	4 (3-4)	0.564	0.851	0.837
Noise	3 (3-3)	4 (4-4)	<0.001	3 (3-4)	4 (4-4)	0.001	0.650	0.833
Edge Sharpness	3 (3-4)	4 (4-4)	0.003	3 (3-4)	4 (4-4)	0.003	0.837	0.837
Diagnostic Confidence	4 (4-4)	4 (4-4)	0.157	4 (4-4)	4 (4-4)	0.180	0.632	0.781
Labrum	4 (3-4)	4 (4-4)	0.059	4 (3-4)	4 (4-4)	0.157	0.803	0.841
Iliofemoral Ligament	5 (4-5)	5 (4-5)	0.157	5 (4-5)	5 (5-5)	0.206	0.896	0.833
Ligamentum Teres	4 (3-4)	4 (4-4)	0.161	4 (3-4)	4 (4-4)	0.366	0.720	0.833
Psoas Tendon	4 (3-4)	4 (3-4)	0.317	4 (3-4)	4 (3-4)	0.655	0.883	0.837
Abductor Tendons	4 (4-4)	4 (4-5)	0.157	4 (4-4)	4 (4-5)	0.157	0.837	0.881
Cartilage	4 (3-4)	4 (4-4)	0.414	4 (3-4)	4 (4-4)	0.655	0.875	0.833

**Table 4:** Results for detection of pathologic findings—comparison between TSE_S_ and TSE_DL_. Only the results of reader 1 are presented because of almost perfect inter-reader agreement. DJD: degenerative joint disease.

	Reader 1	
Pathology	TSE_S_	TSE_DL_
Labrum	11 (78.6%)	11 (78.6%)
Iliofemoral ligament	0 (0.0%)	0 (0.0%)
Ligamentum teres	4 (28.6%)	4 (28.6%)
Psoas tendon	0 (0.0%)	0 (0.0%)
Abductor tendons	4 (28.6%)	4 (28.6%)
Cartilage	4 (28.6%)	4 (28.6%)
DJD	7 (50.0%)	7 (50.0%)
Bursitis	1 (7.1%)	1 (7.1%)
Fracture	0 (0.0%)	0 (0.0%)
Effusion	3 (21.4%)	4 (28.6%)

## Data Availability

The datasets used and analyzed during the current study are available from the corresponding author on reasonable request.

## Abbreviations

Compressed sensing (CS), deep learning (DL), image quality (IQ), interquartile range (IQR), magnetic resonance imaging (MRI), signal-to-noise ratio (SNR), standard (S), turbo spin echo (TSE), three-dimensional (3D), two-dimensional (2D), proton density (PD), parallel imaging (PI).
